# Post-COVID-19 resurgence of scabies’ cases in the Lazio Region, Italy: a new emerging public health threat?

**DOI:** 10.1186/s40249-025-01279-8

**Published:** 2025-02-04

**Authors:** Martina Spaziante, Alessandro Agresta, Maurizio D’Amato, Gabriella De Carli, Gilda Tonziello, Valentina Vantaggio, Giorgio Nicolò Malatesta, Enrico Girardi, Alessandra Barca, Paola Scognamiglio, Francesco Vairo

**Affiliations:** 1 National Institute for Infectious Diseases Lazzaro Spallanzani-IRCCS, Rome, Italy; 2 Health and Social Policy, Lazio Region, Rome, Italy

**Keywords:** Scabies, Epidemiology, Outbreak, Italy

## Abstract

Scabies represents a global health issue and in 2017 was added to the World Health Organization’s list of neglected tropical diseases. In European and Middle Eastern countries, cases are sporadic while recent surveillance data have pointed out an increasing incidence among vulnerable populations. Regional cases for Lazio, Italy, reported from 2017 to 2023 to the national infectious disease surveillance system were analyzed. In Lazio, just after the coronavirus disease 2019 (COVID-19) pandemic onset, a significant and immediate reduction in the incidence of scabies was recorded (− 79.6%) followed by a progressive and relevant increase (143.4% from 2020 to 2021, 142.3% from 2021 to 2022 and 170.3% from 2022 to 2023). Consistently, the number of scabies outbreaks, after a decrease following the first COVID-19 wave, has progressively increased over time, mainly due to the occurrence of outbreaks in long term facilities (750% from 2020 to 2023). The increased incidence may also be driven by the “pseudo-resistance” phenomenon (under dosed/early-discontinued treatment, suboptimal adherence, reduced drug bioavailability), but also by reduced in-vitro susceptibility to the mainly used scabicides. The rapidly evolving epidemiology of scabies in our country, as documented also in other regions, calls for a comprehensive approach to effectively address the problem.

Scabies is a parasitic disease caused by the mite *Sarcoptes scabiei* that can lead to secondary skin infections (e.g. impetigo). The disease represents a global health issue and in 2017 was added to the World Health Organization’s list of neglected tropical diseases. In 2020 scabies was estimated to be responsible for 4.84 million disability-adjusted life years and 565 million (499–634) incident cases worldwide [1]. In European and Middle Eastern countries, cases are sporadic and the prevalence of the disease in the general population is low (< 2%), perhaps due to socioeconomic factors and climatic conditions. Consequently, in these regions the disease is not generally perceived as a public health concern [2].

However, recent surveillance data have highlighted an increasing incidence of scabies in some European countries, especially among vulnerable populations, such as immunosuppressed/long-term care patients, migrants/refugees or prisoners [3]. Furthermore, the coronavirus disease 2019 (COVID-19) emergency has represented a profound disruption in all infectious disease transmission dynamics worldwide.

To assess the trajectories of scabies epidemiology in the Lazio Region, Italy, regional cases reported from 1 January 2017 to 31 December 2023 were extracted from the databases of the two national infectious disease surveillance systems successively in force (Sistema Informativo Malattie Infettive—Infectious Disease Information System: 2017–2019; and the new national Infectious Disease Reporting System PREMAL: 2020–2023). Both collect a common core dataset across all ages and allow linking epidemiologically-related cases occurring in public and private settings: home, school, school-camp/summer-camp, sporting facilities, hotel, workplace, military base, prison, refugee centers, nomad camps, rehabilitation centers for drug addicts, hospital, long-term care facilities, mental institution.

The annual incidence of notified scabies cases in the Lazio Region, Italy, per 100,000 residents is shown in Fig. 1.

**Fig. 1:**
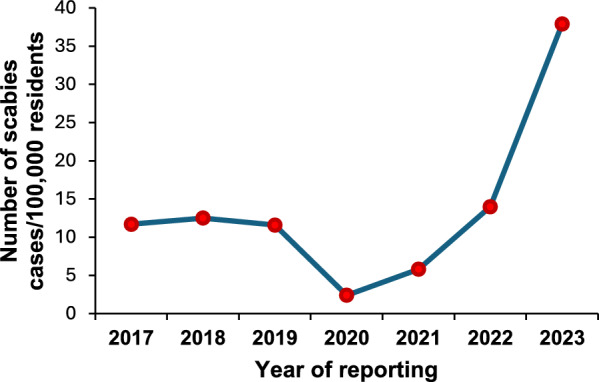
Annual incidence per 100,000 residents of scabies cases reported in the Lazio Region, Italy, from 1 January 2017 to 31 December 2023 by year of reporting (data updated to 6 October 2024)

As shown, in the first year of the COVID-19 pandemic there was a significant and immediate reduction in the incidence of scabies (− 79.6%) (Fig. 1). Subsequently, starting from 2021 and, more significantly, during the COVID-19 recovery phase, the annual incidence of scabies has shown a progressive and significant increase (143.4% from 2020 to 2021, 142.3% from 2021 to 2022, and 170.3% from 2022 to 2023).

Consistently, the number of scabies outbreaks, after a decrease following the first COVID-19 wave, has progressively increased over time, mainly due to the occurrence of outbreaks in long-term care facilities (i.e. rehabilitation centers, nursing homes, residential long-term care), which recorded an increase of 750% from 2020 to 2023 (Fig. 2).

**Fig. 2:**
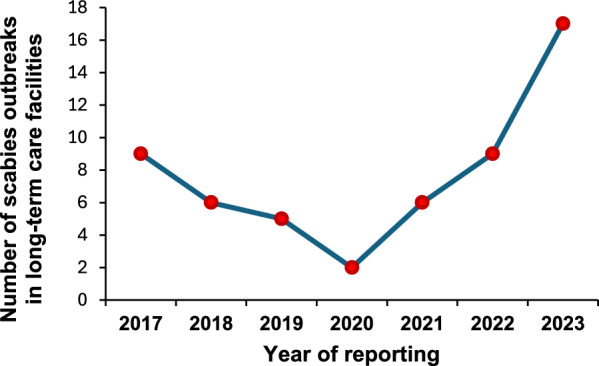
Annual number of scabies outbreaks involving cases which recognize a long-term care facility as possible place of exposure, reported in the Lazio Region, Italy, from 1 January 2017 to 31 December 2023, by year of reporting (data updated to 6 October 2024)

In our view, the decrease in incidence recorded in 2020 was at least partly due to the negative impact of COVID-19 on reporting activities, resulting from the shift in public health efforts to combat the pandemic. On the other hand, the subsequent growth in incidence, which began to be evident since 2021, could be driven by several circumstances.

First, the COVID-19 pandemic has led to a high turnover of beds in COVID-19 wards (for hospital cases) and a shortage/lack of specialized nursing staff, increasing the risk of admission of patients with undiagnosed scabies, and crowding of long-term care facilities (where many hospital cases were also admitted post-COVID, or where COVID-19 cases with undiagnosed scabies were originated and admitted to hospital). For household-transmitted cases, prolonged and close contact with cohabitants following ‘stay‐at‐home’ policies has favored transmission [4], in addition to already constituting a challenging diagnosis for general practitioners and pediatricians.

Second, the increasing incidence may depend at least in part on increasing rates of treatment failure, mainly driven by the phenomenon of “pseudo-resistance” (under-dosed/early discontinued treatment, suboptimal adherence -especially in elderly/psychiatric patients-, reduced drug bioavailability), but also by a reduced in-vitro susceptibility to the mainly used scabicides (permethrin and ivermectin) [3]. Indeed, a growing body of clinical and in-vitro evidence suggests increasing resistance to permethrin, while, on the other hand, resistance to benzyl-benzoate is anecdotal, despite long-standing use.

In conclusion, our results could suggest that the epidemiology of scabies is rapidly evolving in Italy, as confirmed also in other regions [5]. To effectively address the problem, a comprehensive approach is essential, including enhanced diagnostic efforts, intensified infection control measures in at-risk settings, and public health strategies that address the socioeconomic factors underlying the spread of scabies, including policies to combat stigma. Furthermore, innovative therapeutic strategies (combination regimens, reintroduction of older therapies or development of new compounds) are needed, possibly reconsidering the role of permethrin as a first-line treatment option [3].

## Data Availability

The data that support the findings of this study are not openly available due to reasons of sensitivity and are available in aggregated form from the corresponding author upon reasonable request. Data are located in controlled access data storage at the Italian Ministry of Health.
